# MicroRNAs in melanoma development and resistance to target therapy

**DOI:** 10.18632/oncotarget.14763

**Published:** 2017-01-19

**Authors:** Luigi Fattore, Susan Costantini, Debora Malpicci, Ciro Francesco Ruggiero, Paolo Antonio Ascierto, Carlo M. Croce, Rita Mancini, Gennaro Ciliberto

**Affiliations:** ^1^ Istituto Nazionale per lo Studio e la Cura dei Tumori “Fondazione G. Pascale”, Napoli, Italia; ^2^ CROM, Istituto Nazionale Tumori “Fondazione G. Pascale”-IRCCS, Napoli, Italia; ^3^ Dipartimento di Medicina Sperimentale e Clinica, Università degli Studi di Catanzaro “Magna Graecia”, Catanzaro, Italia; ^4^ Department of Molecular Virology, Immunology, and Medical Genetics, The Ohio State University Comprehensive Cancer Center, Columbus, OH, USA; ^5^ Dipartimento di Medicina Clinica e Molecolare, Sapienza Università di Roma, Roma, Italia; ^6^ IRCCS Istituto Nazionale Tumori “Regina Elena”, Roma, Italy

**Keywords:** melanoma, miRNA, target therapy, drug resistance, intracellular pathways

## Abstract

microRNAs constitute a complex class of pleiotropic post-transcriptional regulators of gene expression involved in the control of several physiologic and pathologic processes. Their mechanism of action is primarily based on the imperfect matching of a seed region located at the 5′ end of a 21-23 nt sequence with a partially complementary sequence located in the 3′ untranslated region of target mRNAs. This leads to inhibition of mRNA translation and eventually to its degradation. Individual miRNAs are capable of binding to several mRNAs and several miRNAs are capable of influencing the function of the same mRNAs. In recent years networks of miRNAs are emerging as capable of controlling key signaling pathways responsible for the growth and propagation of cancer cells. Furthermore several examples have been provided which highlight the involvement of miRNAs in the development of resistance to targeted drug therapies. In this review we provide an updated overview of the role of miRNAs in the development of melanoma and the identification of the main downstream pathways controlled by these miRNAs. Furthermore we discuss a group of miRNAs capable to influence through their respective up- or down-modulation the development of resistance to BRAF and MEK inhibitors.

## INTRODUCTION

Malignant melanoma is a neoplasm of melanocytes and its incidence has increased dramatically over the past few decades [1]. Surgery is still the main and definitive treatment for early-stage melanoma, but it is rarely curative for the advanced stages of melanomas [1]. Chemotherapy represents the past for the treatment of metastatic melanoma and it was based on 2 FDA-approved drugs: fotemustine, dacarbazine [2]. In recent years, however, advances in the use of immunotherapy and targeted therapy have revolutionized clinical history of this disease thanks to their ability to significantly enhance proportion and duration of objective responses and to provide extended prolongation of patients’ survival. Immunotherapy is mostly based on immune checkpoint inhibitors targeting CTLA4 and, more recently, PD1/PDL1 interaction [3]. Targeted therapies with MAPK pathway kinase inhibitors (KIs) have been developed thanks to the discovery that BRAF and NRAS mutations are among the major oncogenic drivers of melanoma proliferation and survival [4].

Approximately 50% of patients harbor v-raf murine sarcoma viral oncogene homolog B1 (BRAF) V600 mutations [5]. In 90% of cases BRAF mutations change Valine 600 into glutamic acid (V600E) [5]. Less frequently substitution with other aminoacids (V600D, V600R) is observed [5]. BRAFV600 mutated oncogenes lead to the uncontrolled activation of the mitogen-activated protein kinase (MAPK) signalling pathway and act as the main oncogenic drivers of melanoma progression and proliferation [6]. These evidences lead initially to the clinical development of BRAF inhibitors, such as vemurafenib and dabrafenib, which are selective inhibitors of BRAF-V600 mutated oncogenes and have been approved by FDA [7, 8]. BRAF inhibitors (BRAFi) are active only in melanoma cells bearing V600 BRAF mutations, where this kinase is present as constitutively active monomers. In contrast BRAFi exert a paradoxical tumor promoting effect in RAS mutated melanomas where they induce the allosteric activation of heterodimeric complexes formed by mutated and wild-type BRAF monomers [9]. Single-agent vemurafenib and dabrafenib demonstrated unprecedented objective responses and improvements in progression-free and overall survival in patients with metastatic melanoma bearing BRAF V600E mutation, as compared to old chemotherapy approaches [10]. However, the duration of response was limited in time and the median progression free-survival extended only to 6-8 months because of the development of drug resistance [11, 12].

Acquired resistance to BRAF inhibitors is usually characterized by reactivation of the MAPK pathway [13, 14]. Furthermore, the scenario is complicated by the paradoxical development of secondary skin tumors, which may arise from the BRAF inhibitors-induced activation of MAPK pathway in wild-type BRAF cells [15]. For these reasons more recently the gold standard of therapy for BRAF mutated melanoma has become the combinations of different BRAF inhibitors (such as vemurafenib, dabrafenib and very recently encorafenib) with MEK inhibitors (such as trametinib, cobimetinib or binimetinib) [16–20]. These combinations significantly increase the percentage of objective responses, prolong overall and progression-free survival compared to single-agent therapies and mitigate the emergence of resistance. However, also dual inhibition inevitably fails in the long term in the majority of cases [21, 22].

Several studies have been directed to understand the molecular mechanisms of acquired resistance. The first studies conducted on acquired resistance to BRAFi monotherapy identified NRAS or KRAS *de novo* mutations, mutant BRAF V600E amplification or its alternative splicing, MEK1/MEK2 mutations or CDKN2A loss at the basis of resistance [21, 23–25]. All these molecular alterations converge in the reactivation of the MAPK pathway. Furthermore, Shi and colleagues, through an intensive DNA deep sequencing analysis of a large number of tumor samples from patients resistant to different BRAFi monotherapies, confirmed that mutations correlated to the MAPK pathway are evident in the majority of cases (70%) [26]. Genetic alterations were found also in the PI3K/PTEN/AKT signalling pathway in 22% of cases [26]. The scenario is complicated by the existence of concomitant genetic alterations in both core drug escape pathways in 18% of cases, which occur in the same tumor or among multiple tumors from the same patient [26]. A more recent study also investigated the mechanisms of acquired resistance to BRAF and MEK inhibitors [22]. The analysis by whole exome sequencing, conducted on melanoma tissues from 28 patients suffering of double-drug disease progression, identified in the majority of cases (about 68%) molecular alterations in the MAPK and PI3K/PTEN/AKT signaling pathways, as previously reported [26], i.e. same genetic alterations, which occur in the resistance to BRAFi monotherapies were evident also in the double-drug disease progression [22]. These studies taken together suggest that also hitting hard melanoma cells simultaneously with BRAFi+MEKi combinations does not prevent the activation of escape mechanisms leading eventually to the selection of resistant cells bearing activation of the same survival and proliferation pathways. The question then arises as to which are these escape mechanisms.

In our opinion the answer can be found in a better understanding of adaptive epigenetic and/or post-transcriptional mechanisms of resistance. In a significant percent of cases of drug resistant melanomas (about 26%) no new mutations have been found [26, 27]. Recent studies showed that melanoma cells exposed to MAPK inhibitors undergo early adaptive responses, which help the emergence of drug resistant cells [28, 29]. We and others, for example, identified the rapid phosphorylation of the ErbB3 receptor and the activation of the downstream AKT pathway as a key event responsible for the development of resistance to targeted therapies in melanoma through the activation of a feedback autocrine survival loop involving increased production the ErbB3 ligand neuregulin1 (NRG1) [30, 31]. Moreover, we demonstrated that blocking ErbB3 activity with a combination of neutralizing antibodies not only abolished early adaptive responses, but also impaired the establishment of long-term resistance [30, 32].

We believe that a variety of post-transcriptional adaptive changes orchestrate the development of drug resistance, which involve also non-coding RNAs. In this context since microRNAs are important multifunctional post-transcriptional modulators of gene expression, which play key-roles in various human cancers [33, 34] it is of utmost importance to analyze their involvement in drug resistance. Here we review the emerging role of miRNAs as key players in melanoma progression and development of resistance and discuss the potential diagnostic and therapeutic implications.

## MICRORNAS AS MAJOR POST-TRANSCRIPTIONAL MODULATORS OF GENE EXPRESSION

During the last two decades small non-coding RNAs have been described as the undisputed protagonists of the eukaryotic post-transcriptional machinery regulation [35]. Among them microRNAs (miRNAs) have become the subject of the most intensive studies and nowadays thousands of papers have been published on this matter. Furthermore there are over 2500 known human miRNAs, which are recorded in various online available databases [36]. miRNAs are short RNA about 22 nucleotides long which were found to be the most expressed class of non-coding RNAs in eukaryotic somatic tissues [33]. Their main function is the modulation of gene expression through mRNA silencing or degradation and usually miRNAs have pleiotropic effects because a single miRNA is potentially able to target simultaneously several mRNAs [36, 37]. This feature explains how miRNAs are such a powerful regulators of gene expression and the complexity and multitude of cellular pathways they can affect. miRNAs usually induce the block of translation and the following destabilization of the mRNA target through an imperfect binding to its 3′UTR [36]. The 5′end domain (position 2 to 8) of each miRNAs is responsible for the recognizing and is called “seed region” [38]. Mature miRNAs, which share identical sequences at nucleotides 2-8 are generally considered to belong to the same “miRNA family” [39]. Virtually all mRNAs have conserved or non-conserved miRNAs binding sites and are supposed to be under miRNA post-transcriptional control. Hence, the biogenesis and the regulation of miRNAs themselves are tightly regulated [39]. The locations of miRNA sequences are in various genomic contexts and they are all transcribed, capped and polyadenylated by RNA polymerase II in a long primary transcript, called pri-miRNAs [36, 37]. This long transcript is processed into the nucleus by the Drosha/DGCR8 complex, which chops the pri-miRNAs generating the pre-miRNA 70 bp long [36, 37]. pre-miRNAs are exported into the cytosol where they are processed by the RNAse III Dicer endonuclease originating the mature miRNA duplex [36, 37]. The mature miRNA is ready to be loaded together with the Argonaute2 and the transactivation-responsive RNA-binding proteins to form the RISC complex (RNA-induced silencing complex) [36, 37]. This complex removes and degrades the complementary strand and retains the fully functional miRNA. The perfect or imperfect binding complementary determines the mRNA fate; indeed in the first case it is degraded while in the second case it is inhibited in its translation [38].

miRNAs are classified as “intergenic” or “intronic” or on the basis of their genomic location and are mostly situated in clusters [40]. The first ones are transcribed from their own promoters, while the second ones are encoded by introns of noncoding or coding transcripts and thus share the promoter with their host gene expression [40]. Human miRNA gene clusters are generally co-transcribed, but the individual mature miRNAs can also be regulated at post-transcriptional levels [39]. Several known transcription factors, for example p53, MYC, ZEB1 and ZEB2, have been described as positive or negative regulators of miRNA expression [39]. Furthermore, miRNA regulation can be exerted also by epigenetic control through DNA methylation and histone modifications. miRNA regulation at post-transcriptional level is carried out through various mechanisms, such as RNA “tailing” through the addition of untemplate nucleotide at the 3′ end or adenylation, through RNA methylation or regulation of RNA stability by several specific nucleases [39]. miRNAs dysregulation plays a key-role in various human diseases, especially in cancer [34]. Of note, different tumor types have specific miRNA signatures compared not only to healthy tissues but also to other cancers.

Calin and colleagues were the first to report in 2002 miRNA deregulation in human cancer identifying miR-15a/16-1 cluster deletion in chronic lymphocytic leukemia [41]. This deletion induces the overexpression of the anti-apoptotic B-cell lymphoma 2 (BCL2), which is a target of these miRNAs [41]. Furthermore, the same investigators demonstrated that more than half of the known miRNA are located in genomic regions whose alteration is frequently reported in human cancers [42]. Nowadays several cancer-associated miRNAs are known, which are described to act as oncosuppressor miRNAs or oncomiRs. Examples of the first ones are: miR-15/16, let-7, miR-200, miR-34, miR-107 and miR-126, whereas examples of the second ones are: miR-221/222, miR-21 and miR-10b [37].

## MICRORNA DEREGULATION IN MELANOMA DEVELOPMENT AND PROGRESSION

In the last years several studies have analyzed the involvement of miRNA in the progression of metastatic melanoma [43–53]. We will summarize here below the findings of a number of representative studies.

Mueller and colleagues were the first to study differential miRNA expression between melanocytes and melanoma cell lines through a microarray-based profiling [43]. Using stringent criteria (up- or down-regulation more than 10-fold) they identified 63 miRNAs deregulated (49 up-regulated and 14 down-regulated) in primary melanomas *vs* normal melanocytes, which were considered to be associated to the early progression of melanoma. In a second type of analysis the same authors compared the miRNAome of HMB2 melanoma cells before and after stable transfection of an antisense melanoma inhibitory activity (MIA) construct. Here they observed a reverse trend, namely 12 up- and 34 down-regulated miRNAs respectively. Comparison of the first with the second list revealed 18 miRNAs inversely regulated, namely upregulated in early progression and downregulated when MIA was knocked down and viceversa. In a third type of analysis comparison of miRNA expression between primary melanomas and metastatic samples led to the identification of a smaller set of dis-regulated miRNA, namely 11 up-regulated and 2 down-regulated which were thus considered to be involved in metastatic colonization of melanoma cells.

A similar study was carried out by Caramuta et al [44], who compared miRNA expression profiles in clinical samples of melanoma metastasis, melanoma cell lines and normal melanocyte cultures. They focused on 167 miRNAs, which were clustered in relation with clinical characteristics, patient survival, and mutational status for BRAF and NRAS [44]. These researchers, performed two types of analysis to identify miRNAs deregulated in melanomas *vs* melanocytes. A significant analysis of microarray (SAM) analysis identified 32 most important deregulated miRNAs, of which 13 overexpressed and 19 down-regulated. In addition the Prediction Analysis of Microarrays (PAM) method revealed a minimal miRNA signature composed of 10 miRNAs capable to distinguish the two groups with a prediction accuracy of 100%. Of note, among these miRNAs, miR-126 had been identified in the previous study by Mueller et al [43]. Furthermore, applying the same PAM analysis Caramuta et al. identified six candidates miRNAs that could predict disease outcome [44]. Among them two were validated by RT-PCR, miR-191 and miR-193b. High expression of miR-191 was confirmed to be highly correlated with better survival, while high expression of miR-193b was confirmed to correlate with poorer survival respectively [44].

Chan et al analyzed the expression of a panel of 384 miRNAs between 42 patient derived primary melanomas samples with three normal melanocyte control samples and found eight miRNAs to be differentially expressed [45]. Of these eight, two, miR-183 and miR-135b are present in the signature identified by Muller et al [43], while other two, namely miR-132 and miR-211 in the signature by Caramuta et al [44]. The same authors observed that miRNA expression profiles are able to distinguish among melanoma subtypes such as acral *versus* non non-acral melanomas [45]. Among their most interesting observations was that out of 32 non-acral melanoma samples genotyped for a KRAS oncogene variant located in the 3′ untranslated region (3′UTR) and known to be affecting the binding site for miRNAs, 25% were positive for the KRAS-variant, thus suggesting the association of this variant with the increased risk of developing melanomas. When they compared the KRAS-variant group and the non-KRAS-variant one, they found the significant down-regulation of only one miRNA, miR-137 [45]. Of notice, among the genes reported to regulate or to be regulated by miR-137 the most relevant is micropthalmia-associated transcription factor (MITF) [54].

In addition to large profiling data above described some specific miRNAs have been the object of further investigations during the last years. Let-7a, for example, which is a member of a known family of oncosuppressor microRNAs, has been reported to be downregulated in melanoma cells compared to melanocytes [55]. Moreover, this miRNA targets human integrin β_3_, which has a well-known role in melanoma progression and invasion. Through experiments of transient *in vitro* overexpression and luciferase assays, was it possible to link the up-regulation of integrin β_3_ occurring in melanoma cells to the amount of let-7a present in the cell, thus demonstrating that this miRNA has an oncosuppressive role in melanoma [55]. Moreover recent findings have attributed to this miRNA a key role in regulating energy metabolism in cancer cells by mediating mitochondrial ROS production with the concomitant up-regulation of oxidative stress responsive genes [56]. Another miRNA, which specifically controls melanoma cell invasion is miR-339-3p. This miRNA has been identified by a comprehensive functional screen of a human miRNA mimetic library in a cell-based assay for invasion [57]. This miRNA targets myeloid cell leukemia sequence 1 (MCL-1), a well-known oncogene in several human malignancies, including melanoma [58]. In addition miR-339-3p enforced expression is able to affect melanoma cell invasion not only *in vitro*, but also in a model of lung colonization in T-cells deficient mice [57]. Moreover our group very recently identified a novel oncosuppressive miRNA in metastatic melanoma, miR-579-3p [59] whose involvement in melanoma progression and development of drug resistance will be described in detail below.

Conversely the following human poly-cistronic miRNA clusters have been considered to play oncogenic roles in melanoma. The miR-17/92 cluster, for example, codes for miR-17, which has been shown to increase the motility of melanoma cells. miR-17 targets ETV1, which belongs to the ETS (E-twenty six) transcription factor family, which has a suppressive role in melanoma [60]. mir-514a is a member of a cluster of miRNAs (miR-506-514), which are involved in initiating melanocyte transformation and promotion of melanoma growth [61]. miR-146a plays a dual role in melanoma malignancy. Its upregulation during melanoma progression triggers tumor growth through inhibition of lunatic fringe (LFNG) and NUMB and activation of the NOTCH/PTEN/AKT pathway. In contrast its downregulation in Circulating Tumor Cells (CTC), suppresses tumor dissemination through modulation of the expression of ITGAV and ROCK1 [62, 63]. miR-638 is able to promote melanoma metastasis since it is overexpressed in metastatic lesions compared to primary melanomas [64]. This miRNA downregulates TP53INP2 oncosuppressor thus protecting melanoma cells from apoptosis and autophagia [64]. Lastly, Felicetti et al [65] showed that miR-222, a well-known oncogenic miRNA, which is encoded together with miR-221 [66], drives melanoma development and dissemination. miR-222 is transported in exosomes to drive melanoma malignancy from miR-overexpressing cells to the recipient primary melanoma cells [65]. Furthermore it was possible to correlate miR-exosomal expression to the reduction of known miR-222 target genes, such as p27, and conversely to the induction of the PI3K/AKT pathway, thus confirming its functional implication in melanoma development, as like as in other human cancers [65].

## ONCOGENIC BRAF-V600 AND MAPK SIGNALING ACT IN CONCERT WITH MIRNA DEREGULATION TO AFFECT MELANOMA DEVELOPMENT

The RAS-RAF-MAPK signaling pathway has a central role in sustaining the oncogenic and proliferative phenotype of melanoma cells, as well as in other human cancers [67]. For this reason, miRNA involvement in the regulation of this pathway has been the focus of intensive research efforts. Let-7 was the first miRNA to be described to directly target KRAS, one of the most important members of MAPK pathway, frequently mutated in human cancers [68]. Furthermore, polymorphisms in KRAS 3′UTR alter let-7 binding, thus resulting in the aberrant activation of the MAPK pathway in non-small cell lung cancer [68]. On the reverse MAPK activity results in the aberrant expression of a subset of miRNAs in pancreatic cancer cells. Four miRNAs: miR-7-3, miR-34a, miR-181d, and miR-193b, have been preferentially associated with MAPK activity [69]; in particular that the promoter of host genes for miR-7-3 and mir-34a are both downregulated by the constitutive activation of MAPK [69]. Therefore there seems to be a reciprocal capability of selected miRNAs to regulated of MAPK pathway and viceversa.

The most thorough analysis of the relationship between the oncogenic BRAF signaling and miRNAs expression and function in metastatic melanoma was carried out by Couts at al [70]. The high relevance in melanoma of oncogenic BRAF-V600 mutations, which occur in approximately 50% of cases and their impact on the constitutive activation of the MAPK pathway provided a strong rationale for this study. First of all miRNA expression levels were compared between melanocytes and six BRAF-mutated melanoma cell lines through a sensitive microarray profiling platform [70]. This led to the identification of more than 30 miRNAs differing between melanocytes and melanoma cells. Most of them had not been previously described in melanoma, but were known to be oncogenic or oncosuppressive in other human cancers. Among them, for example, miR-17-92 was known to be associated to tumor progression in lung, colon cancer, B-cell lymphoma and glioblastoma [71]; whereas miR-34a and let-7 family members are known oncosuppressor miRNAs in lung, breast, prostate, gastric cancer, pancreatic cancer and neuroblastoma [72, 73]. When miRNAs expression was analyzed in the same melanoma cell lines before and after treatment with MEK inhibitors a reciprocal miRNAs deregulation was observed in more than half of the cases, thus confirming the correlation between miRNA deregulation and activation of the MAPK pathway [70]. In order to provide functional insights on the deregulated miRNAs, the authors transfected them in melanoma cells and observed that 14 of them were able to affect cell proliferation [70]. Seven miRNAs, which were reduced by BRAF/MAPK signaling pathway, such as let-7i, miR-34a and miR-22 effectively inhibited melanoma cell proliferation. On the reverse, seven other miRNAs, including miR-17 and miR-92/95, which were up-regulated by BRAF/MAPK signaling pathway were able to increase cell proliferation. Investigators then proceeded to identify the molecular pathways and mRNAs affected by the deregulated miRNAs and were able to demonstrate through transient transfection and luciferase experiments that BRAF/MAPK regulated miRNAs converge on a complex combinatorial control of a specific set of key cancer regulatory genes involved in cell cycle/proliferation, adhesion/invasion, signaling and survival [70]. It was thus possible to conclude that mutated BRAF exerts multiple regulatory effects on melanomagenesis through the induction or the repression of a complex network of miRNAs whose targets are responsible for the promotion or the suppression respectively of melanoma growth. However, how this network of miRNAs is regulated by the constitutive activation of the MAPK pathway still remains an open question, which will require further investigation. It has to be added that the association of BRAF mutational status with miRNA deregulation was also confirmed in other human cancers. For example in colorectal cancer (CRC) it was observed that miR-31 is up-regulated in V600E-BRAF mutated CRCs compared to wild-type CRCs and this is related to cancer-specific mortality [74]. As mentioned above the MAPK pathway can be the reciprocal target of specific microRNAs. This is the case of miR-340. Notably miR-340 is able to affect the expression of 39 out of 45 total RAS-RAF-MAPK components, such as BRAF, KRAS, NRAS and MYC, thus reducing melanoma cell growth and migration [75]. Furthermore Liu and colleagues, through a screening using a high-throughput quantitative real-time miRNA PCR array, identified miR-524-5p as downregulated in BRAF mutated melanoma cells but not in wild-type BRAF cells [76]. miR-524-5p targets both BRAF and ERK2 genes, which are the key regulators of the MAPK pathway. Furthermore, the same authors demonstrated that miR-enforced expression is able to affect melanoma cell migration and proliferation both *in vitro* and *in vivo*, thus confirming its effect on the inhibition of these two potent oncogenic members of the MAPK pathway [76].

**Figure 1:**
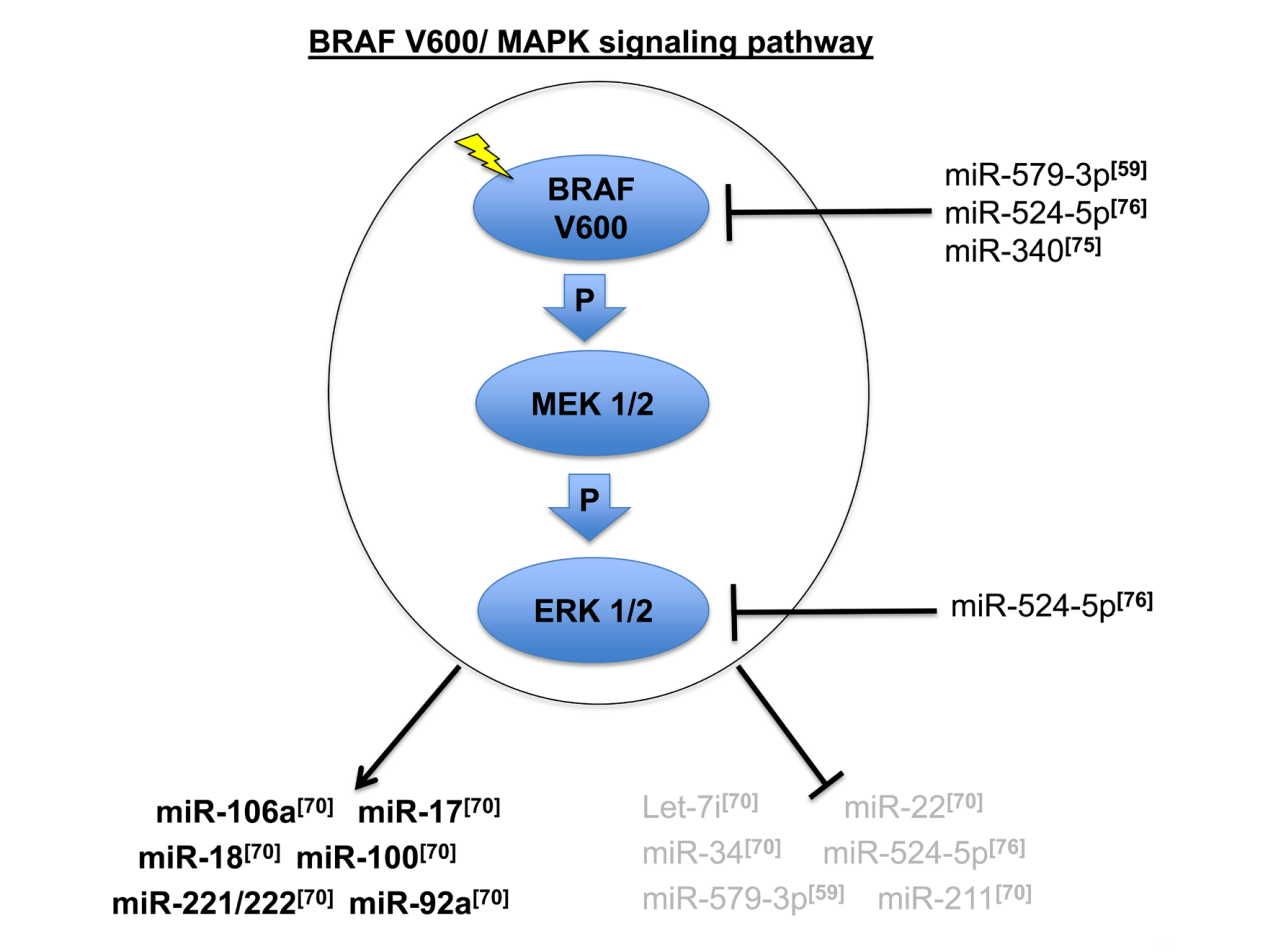
Oncogenic BRAF V600/MAPK signaling pathway and the most relevant miRNAs connected to its deregulation in metastatic melanoma [59, 70, 75, 76] Arrows and blocking bars indicate respectively the positive or negative regulation of MAPK signaling on a sets of miRNAs. Blocking bars also indicate the repression of specific miRNA exerted on specific members of this signaling pathway.

In conclusion, deregulation of the MAPK pathway and miRNA epigenetic control strongly influence tumor cell fate disrupting normal homeostasis in tumors addicted to this pathway. We represent in the diagram in Figure 1 the most relevant miRNAs identified by the studies described above, which are regulated or which regulate the oncogenic BRAF V600/MAPK signaling in melanoma.

## DEREGULATED MIRNAS CONVERGE ON A SET OF KEY INTRACELLULAR PATHWAYS

Both comprehensive miRNAome profiling studies [43–53, 70] as well as individual miRNAs analyses [55–65] described above have shown that deregulation of several miRNAs contributes to the development of melanoma. The emerging picture is rather complex because these studies identified different subsets of miRNAs, thus mirroring the great degree of heterogeneity of these tumors. However, since it is known that different miRNAs can modulate the activity of the same mRNAs or also of different mRNAs coding for members of the same signaling pathways, we decided to assessed whether the different miRNA signatures observed in different studies correspond to the involvement of the same key signaling pathways. To test our this hypothesis we decided to analyze in a greater detail the relationship among the miRNAs identified in the studies described in the previous paragraphs.

**Figure 2:**
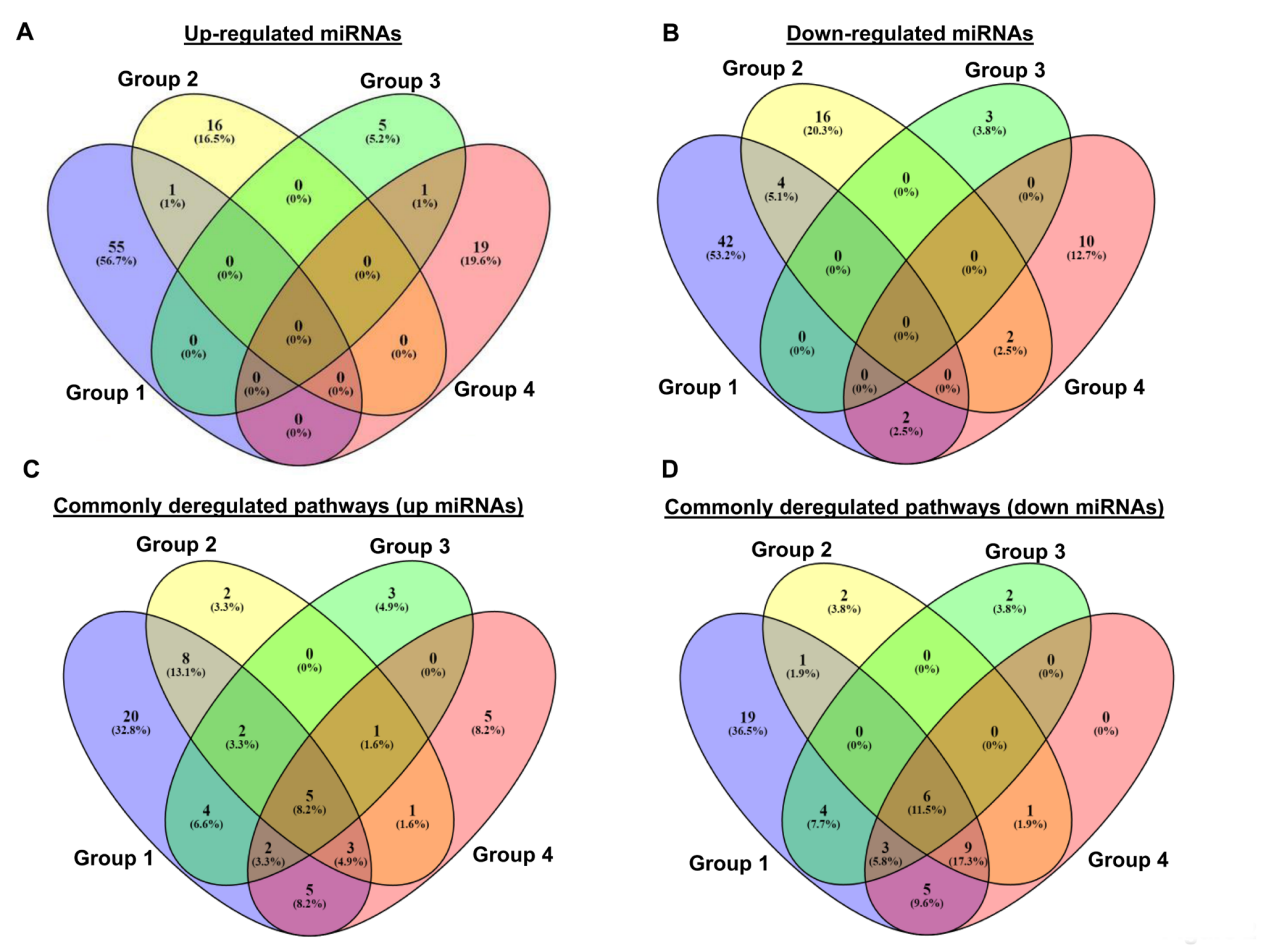
**Comparison between the miRNAs resulted to be in common among the groups 1-4 subdivided between up-regulated A. and down-regulated B. miRNAs, and between the related deregulated pathways C. and D. by Venn diagram (**http://bioinfogp.cn**b.csic.es/tools/venny/).** We show the group 1 in violet, group 2 in yellow, group 3 in green, and group 4 in pink.

Table 1 lists the most representative deregulated microRNAs in melanoma subdivided on the basis of the different studies analyzed: i) Group 1 - miRNA signature associated with early progression of melanoma by a in the large profiling approach described by Muller et al [43]; ii) Group 2 - miRNAs deregulated in melanomas *vs* melanocytes by Caramuta et al and Chan et al. [44, 45]; iii) Group 3 -miRNAs resulted to be deregulated by studies involving individual miRNAs [55–65]; iv) Group 4- miRNA signature associated specifically with BRAF activation and identified by Couts et al [70]. In Table 1 underlined miRNAs are those with possible prognostic and/or diagnostic role in melanoma as reported in some published studies [77, 78]. As shown in Figure 2A and 2B, no miRNAs resulted to be in common among the four groups; we found only one upregulated miRNA (miR-126) in common between groups 1 and 2, one upregulated miRNA in common between groups 3 and 4 (miR-222), four down-regulated miRNAs (miR-196a, miR-374, miR-454-3p and miR-324-5p) in common between the same groups 1 and 2 and two downregulated miRNAs in common between groups 2 and 4 (let-7i and miR-211).

**Table 1:** Most representative deregulated microRNAs in melanoma

	Up-regulated miRNAs	Down-regulated miRNAs
Group 1 Melanoma early progression [43].	miR-9*; miR-10a*; miR-10b*; miR-18a*; miR-26b*; miR-27b; miR-30a-3p; miR-34c; miR-92b; miR-126*; miR-135b; miR-141; **miR-145;** miR-181a; **miR-182**; miR-183; miR-196b; miR-200a; miR-200c; miR-218; miR-224; miR-301; miR-326; miR-335; miR-340; miR-373; miR-379; miR-382; miR-383; miR-449; miR-454-5p; miR-485-3p; miR-504; miR-507; miR-517; miR-518a; miR-518f; miR-520b; miR-520d*; miR-525; miR-526b; miR-539; miR-545; miR-550; miR-557; miR-564; miR-583; miR-606; miR-609; miR-622; miR-628; miR-640; miR-641; miR-658; miR-662; miR-758	miR-21*; miR-23b; miR-126*; miR-141; miR-146a*; miR-148a; miR-148b; miR-181a; miR-196a*; miR-203; miR-299-3p; miR-331; **miR-363**; miR-324-5p*; miR-345; miR-373; miR-374*; miR-422b; miR-449; miR-454-3p*; miR-455; miR-485-3p; miR-487a; miR-489; miR-503; **miR-506**; miR-507; miR-514*; miR-518d; miR-527*; miR-545; miR-550; miR-551b; miR-565; miR-571; miR-577; miR-583; miR-595; miR-596; miR-625, miR-627; miR-632; miR-641; miR-658; miR-660; miR-767-5p; miR-768-3p*; miR-769-5p
Group 2 Melanomas vs melanocytes [44, 45].	let-7b; let-7c; miR-95; miR-126*; miR-132; miR-198; miR-199a; miR-202; miR-210; miR-211; miR-320; miR-423; miR-494; miR-514*; miR-652; miR-765; miR-801	let-7i*; miR-9*; miR-10a*; miR-10b*; **miR-15b;** miR-26b*; miR-30e-3p; miR-98; miR-132; miR-192; miR-194; miR-196a*; miR-211*; miR-324-5p*; miR-374*; miR-454-3p*; **miR-509**; miR-582; miR-602
Group 3 Single miRs with functional involvement [55-65].	miR-17; miR-221; miR-222*; miR-146a*; mir-514a; miR-638	Let-7a; miR-339-3p; **miR-579-3p**
Group 4 Associated to BRAF signaling [70].	miR-7; **miR-17-5p**; miR-18a*; miR-20a; miR-21*; **miR-92**; miR-99a; miR-100; miR-106a; miR-125b; miR-201; miR-212; miR-222*; miR-302c; miR-487b; miR-488; miR-520d*; miR-527*; miR-575; miR-594	let-7g; let-7i*; miR-22; miR-26a; miR-30b; miR-30c; miR-30d; miR-34a; miR-125a; miR-146a*; miR-146b; miR-211*; miR-768-3p*

Group 1: miRNAs associated with early progression of melanoma by a miRNAome profiling approach and selected with at least three-fold up- or down- regulation [43]; Group 2:miRNAs deregulated in melanomas vs melanocytes by two different miRNAome profiling approaches and selected with FDR (false discovery rate) of 0% [44] and with at least three-fold up- or down-regulation [45], respectively; Group 3: miRNAs associated to melanoma by studies involving individual miRNAs [55–65]; Group 4: miRNAs regulated by oncogenic BRAF and selected with at least two-fold up- or down-regulation [70]. Underlined miRNAs have been validated by qRT-PCR. miRNAs in bold have potential diagnostic and/or prognostic values in melanoma based on literature data [77, 78]. miRNAs indicated with an asterisk (*) are in common by at least two groups.

In order to verify if the deregulated miRNAs, reported in Table 1, are able to target same mRNAs and/or signaling pathways, we predicted the putative target mRNAs for all these miRNAs, subdivided in the four groups indicated above, using three online available tools, a) TargetScanHuman [79], b) PITA [80], and c) Miranda [81]. We applied to our analysis stringent criteria, by only selecting mRNAs predicted in common among all three tools. Then, an enriched functional analysis of pathways was performed by the Database for Annotation, Visualization and Integrated Discovery (DAVID) [82]. The results are shown in Supplementary Figure 1. Interestingly, we observed a very high overlap among the mRNAs targets of up- and down-regulated miRNAs [43-45; 55-65, 70]. Most importantly, we identified a number of commonly deregulated pathways where these targets are involved (Figure 2C and 2D). In particular, concerning pathways related to targets of up-regulated miRNAs we observed (Figure 2C) that five pathways are common among the four groups. Looking at pathways related to targets down-regulated by miRNAs (Figure 2D), we found that six pathways are common among all four groups. Of note frequently deregulated pathways are Wnt signaling and other cancer related pathways such as, axon guidance, endocytosis, melanogenesis and calcium signaling (Table 2). The involvement of these pathways in melanoma development and progression has been abundantly reported in the literature [83–92]. Wnt signaling, for example, plays a key role in melanoma development through β-catenin physiological regulation of epidermal melanogenesis [84]. Melanoma cells, indeed, exhibit up-regulated melanogenesis and defective melanosomes [85]. Another example of deregulated pathway is represented by axon guidance, a key element in the formation of neuronal network guided by specific receptors, called plexins [89]. Interestingly both plexins and their guidance molecules semaphorins are lost during melanoma progression [90, 91]. Most importantly plexins are known to be down-regulated by oncogenic BRAF V600 in metastatic melanoma [92], thus confirming the relevance of this pathway underlined by our analysis.

**Table 2:** Common deregulated pathways related to up or down-regulated microRNAs (Groups 1-4)

Common Pathways between:	Pathways related to targets of up-regulated miRNAs	Pathways related to targets of down-regulated miRNAs
Group 1, 2, 3 and 4	- Axon guidance - Wnt signaling pathway - Endocytosis - Regulation of actin cytoskeleton - Pathways in cancer	- Axon guidance - Calcium signaling pathway - Endocytosis - Melanogenesis - Pathways in cancer - Wnt signaling pathway

List of the commonly deregulated pathways related to targets of up-regulated and down-regulated miRNAs belonging to the groups 1-4, reported in Figure 2C and 2D.

In summary, our analysis demonstrates that, while in different melanoma samples and cell lines there is a limited or no overlap in the number of individual miRNAs upregulated or downregulated, on the contrary the same miRNAs converge on a constant set of metabolic pathways, which are key to the development of malignancy.

## MIRNAS INVOLVEMENT IN RESISTANCE TO TARGETED THERAPIES IN BRAF-MUTATED MELANOMAS

Therapy of metastatic cancer is mostly based on the use of chemotherapy and targeted therapies [93]. However, durable objective and clinical responses to these therapies are plagued by the development of chemoresistance which leads to disease relapse in virtually all metastatic patients. Although this scenario is changing in recent years due to the advent of immunotherapy, it has to be stressed that immunotherapy is effective only in a subset of patients. Therefore major efforts are still directed to understand the molecular basis of drug resistance and to develop combination therapies capable to revert acquired resistance or, even better, to avoid the development of resistance. In this context it is of interest to observe that growing evidences point to miRNAs as key factors controlling the emergence of drug resistance.

Garofalo et al. for example, first showed that the oncogenic miR-221/222 induce TRAIL resistance in aggressive non-small cell lung cancer through the activation of the AKT pathway, by targeting PTEN and TIMP3 tumor suppressors [94]. Furthermore, the same group was able to show that the MET oncogene is involved in miR-221/222 activation and that miR-130a, by targeting MET, reduces TRAIL resistance in NSCLC cells through down-regulation of miR-221/222 [95]. Other investigators, demonstrated that miR-19 is involved in the regulation of multidrug resistance (MDR) through the inhibition of the oncosuppressor gene PTEN, thus increasing breast cancer cells resistance to chemotherapeutic agents [96]. Most important miRNAs [61, 59, 94–104, 106, 108] with an impact on the establishment of drug resistance in human cancers are reported in Table 3 and 4. Coming back to metastatic melanoma only few studies have been published about the relationship between miRNAs and drug resistance especially in the context of BRAFi/MEKi based targeted therapies. miR-214, for example, is supposed to play a role in chemoresistance because it has been shown to reduce melanoma cells apoptosis by facilitating survival in adhesion-lacking (anoikis) conditions [105] and also because it is able to modulate drug resistance in many other human cancers, such as lung, ovarian, breast and cervical cancers [106]. However, until now, no study of miR-214 involvement in melanoma drug resistance has been published. In this regard it is important to point out that some miRNAs, such as miR-638 and miR-579-3p, have been also described to affect melanoma cell apoptosis alone or in presence of BRAF inhibitors treatments [59, 64].

**Table 3:** microRNAs facilitators of drug resistance in human cancers

miR facilitators of resistance	Cancer	Targets	Mechanisms	References
miR-34a	Melanoma	NS	Evasion of TRAIL-induced apotosis	108
miR-100	Melanoma	NS	Evasion of TRAIL-induced apotosis	108
miR-125b	Melanoma	NS	Evasion of TRAIL-induced apotosis	108
miR-214	Lung Breast Ovarian Cervical	PTEN	Activation of the PI3K/AKT pathway	106
miR-221/222	Lung Liver	PTEN TIMP3	Evasion of TRAIL-induced apotosis	94, 95
miR-19	Breast	PTEN	Regulation of multidrug resistance	96
miR-17	Glioblastoma	PTEN	HIF-1α activation	97
miR-27a	Ovarian Lung	PTX RKIP	Drug efflux EMT	98 99
miR-21	Pancreatic	PDCD4 PTEN	Inhibition of apoptosis	100

miRNAs, which act as facilitators of the establishment of drug resistance in human cancers, are listed in the table. Not shown (NS) indicates that in the corresponding studies no information about miRNAs target genes have been mentioned.

**Table 4:** microRNAs antagonists of drug resistance in human cancers

miR antagonists of resistance	Cancer	Targets	Mechanisms	References
miR-514a	Melanoma	NF1	Maintenance of MAPK pathway activation	61
miR-200c	Melanoma	BMI	Upregulation of ABC transporters	112
miR-579-3p	Melanoma	BRAF MDM2	Inhibition of MAPK signalling Apoptosis induction	59
miR-130a	Lung	c-MET	Induction of TRAIL sensitivity	95
miR-126	Lung	VEGFA MRP1	Inactivation of the Akt signaling Drug efflux	101
miR-27a	Gastric	ADR	Drug efflux	102
miR-34a	Prostate Gastric	CDK6 CCND1 BCL2	Cell Cycle Apoptosis induction	103 104

miRNAs which act as antagonists of the establishment of drug resistance in human cancers are listed in the table.

Some miRNAs act as “facilitators” of drug resistance. One such example was provided by Stark et al. who suggested the potential involvement of mir-514a in the modulation of sensitivity to BRAF inhibitors in melanoma cells [61]. These investigators showed that NF1, a known tumor suppressor [107], is a direct target of miR-514a [61]. Both NF1 direct silencing by specific silencing with siRNA, and miR-514a upregulation leading to decreased NF1 levels were able to strongly decrease drug sensitivity in short term “*in vitro”* cell proliferation assays [61]. However this study did not ask the direct question of whether up- or down-regulation of miR-514a expression is capable to affect the development of long term resistance and the emergence of resistant melanoma cell populations. Also no effort was made to determine changes in the expression of miR-514a between “ drug sensitive” *vs* “drug resistant” tumor samples derived from patients.

Very recently, it has been shown that BRAF inhibitors induce adaptive cell response *via* cytokine production with a strong cross-talk with miRNAs deregulation, which act as facilitators of drug resistance. Vergani and colleagues proposed that BRAFi-resistant melanoma cells up-regulate chemokine monocyte chemoattractant protein-1 (CCL2), which in turn activate the expression of miR-34a, miR-100 and miR-125b [108]. These miRNAs activation is orchestrated by HIF1 transcription factor [109], which is also known to regulate CCL2 secretion [108]. In line with these findings miR-34a, miR-100 and miR-125b were found to be up-regulated in BRAFi resistant melanoma cell lines and in the biopsies from patients undergoing vemurafenib treatments [108]. Vergani and colleagues’ data are consistent with previous published studies, which evaluated miR-125b, miR-34a and miR-100 involvement in chemoresistance in other tumors through the targeting of proapoptotic genes [108].

An opposite mechanism of action has been attributed to miR-200c, member of a family of known oncosuppressive miRNA [110]. This miRNA has been proposed to counteract the establishment of drug resistance in melanoma by directly targeting Bmi-1, a critical factor involved in the maintenance of stem cells [111]. miR-200c enforced expression reduced the expression of three members of the ATP-binding cassette (ABC) transporters, which mediate chemoresistance in melanoma, thus increasing both targeted therapies and chemotherapy mediated reduction of melanoma cell growth [111]. Also, the biological effects induced by miR-200c overexpression were phenocopied by BMI inactivation. On the reverse, loss of miR-200c increased Bmi-1 expression, thus leading to downregulation of E-cadherin, uppregulation of N-cadherin, and therefore mesenchymal to epithelial transition (MET), upregulation of ABC transporters and activation of the PI3K/AKT and MAPK survival pathways [112]. Finally miR-200c was shown to be downregulated in melanomas that acquire resistance to BRAF inhibitors compared to pretreatment tumor biopsies, in which its target genes are conversely up-regulated [112].

Finally, our group, which studies the mechanisms responsible for the establishment of drug resistance in melanoma [30, 32, 113], has identified a novel miRNA, miR-579-3p as a master regulator of melanoma progression and drug resistance [59]. This miRNA belongs to the miR-548 family, which is emerging as a new family with oncosuppressive role in several human tumors, including breast, ovarian, brain, lung, colon and cervical cancers [114]. We identified miR-579-3p through the online algorithm miRò, by querying potential miRNA deregulated during melanoma progression in relationship with BRAF mutational status [115]. Of note we found that this miRNA has two seed regions that match the 3′-UTR of BRAF [79, 116]. We confirmed, through luciferase and transient overexpression assays its ability to target miR-579-3p and concordantly with these data the reduction of melanoma cell growth and migration. In addition miR-579-3p targets and inhibits the MDM2 oncoprotein [117]. Moreover, we found that low miR-579-3p expression correlates with worse prognosis in melanoma patients and that its expression is further decreased in BRAFi/MEKi resistant melanoma cells. In this regard we observed that miR-579-3p enforced expression both potentiates BRAF and/or MEKi anti-proliferative effects and impairs the establishment of resistance to BRAF inhibitors in long-term clonogenic assay. Most importantly using matched tumor samples from melanoma patients before and after development of resistance, we observed that miR-579-3p is strongly downregulated in tumor biopsies from the same patient after the development of resistance and that this coincides with a reciprocal regulation of the expression of its target oncogenes BRAF and MDM-2 [59]. We are currently investigating the role of a large network of deregulated miRNAs as major players in the establishment of drug resistance in melanoma through the analysis of the whole miRNome profile during the development of resistance. Our unpublished results highlight a gradual deregulation of a growing number of miRNAs, which rewire a complex network of intracellular pathways.

All together these studies point to the involvement of a number of miRNAs either as promoters or as antagonists of resistance (see Table 3 and 4) and show a complex mechanism of regulation by which during the progression of resistance the first groups undergoes upregulation whereas the second undergoes downregulation. How is this process coordinated and which are the main mechanisms controlling the expression of this network of miRNAs will require further studies.

## CONCLUSIONS

The Cancer Genome Atlas (TCGA) allowed the identification of four genetic subtypes of cutaneous melanoma, BRAF mutant (the most common condition responsible for more than 50% of cases), RAS mutant, NF1 mutant, and Triple Wild-Type [118]. Mutations in each of the major driver genes, BRAF, RAS, and NF1, all contribute to deregulation of the MAPK/ERK pathway, leading to uncontrolled cell growth. As recapitulated in this review genetic changes trigger a complex rewiring of intracellular pathways, which involve as major players several microRNAs. How this is accomplished, i.e. how MAPK/ERK deregulation influences the pattern of microRNA expression remains still largely unexplored. Importantly, when normal expression of specific miRNAs is restored in melanomas, reversion of the malignant phenotype is observed both in *in vitro* and in *in vivo* assays, which demonstrates the central role of miRNA in disease pathogenesis.

miRNA deregulation, albeit heterogeneous in different patients and tumor samples (see Figure 1 and 2), constantly converges in a defined subset of intracellular pathways. Indeed our bioinformatic analysis of profiling data from different studies postulates the involvement of specific pathways such as in particular Wnt, axon guidance and exocytosis. Although this intriguing finding will require further validations in the lab, we believe that identification of pathways commonly deregulated by miRNAs in melanoma may lead to a better understanding of disease evolution and to the discovery of additional targets for therapeutic intervention.

Most important for its therapeutic and diagnostic implications is the increasing evidence that miRNA deregulation is heavily responsible for the development of resistance to target therapies. During the development of resistance melanoma select cells with low expression of selected miRNAs, such as miR-200c and miR-579-3p, two potent oncosuppressors acting apparently on different and complementary pathways [59, 111, 112]. Restoration of their expression potentiates the effect of MAPK pathway inhibitory drugs and impairs the establishment of resistance. While it will be important in the future to dissect the molecular mechanism leading to their downregulation, from a therapeutic perspective our strong hope is on the improvement of robust *in vivo* delivery technologies for miRNA mimics capable to restore their normal expression profile in the tumor [119]. Finally, since miRNAs are very stable in human fluids compared to mRNAs because they could be packaged in exosomes or associated with RNA-binding proteins or lipoprotein complexes, which protect them from degradation [120, 121], deregulated miRNA could be used as successful biomarkers in patient plasma and serum, able to early predict resistance to therapies [122–125].

## SUPPLEMENTARY MATERIALS FIGURE


