# Supplementary material for: MicroRNAs in melanoma development and resistance to target therapy
Source: Oncotarget. 2017 Jan 19;8(13):22262–78. doi: 10.18632/oncotarget.14763 (PMC5400662; doi:10.18632/oncotarget.14763)
Supplement: Supplementary file 1 [file oncotarget-08-22262-s001.pdf]

MicroRNAs in melanoma development and resistance to target therapy

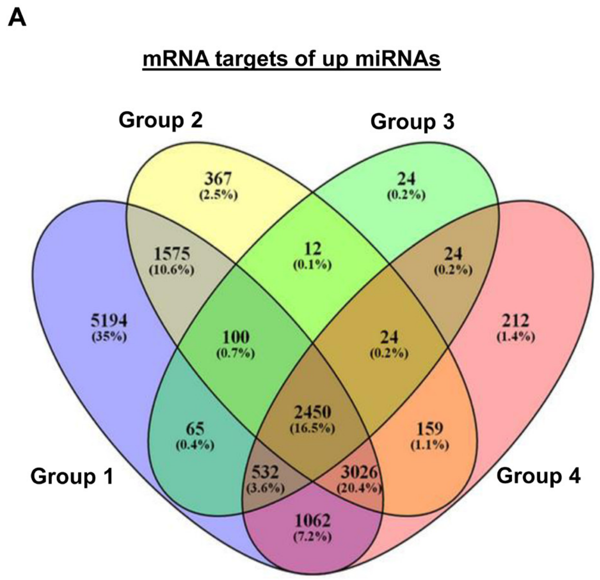

Supplementary Figure 1: Comparison between the putative target mRNAs for all the miRNAs, belonging to groups 1-4, by Venn diagram (http://bioinfogp.cnb.csic.es/tools/venny/). We show the group 1 in violet, group 2 in yellow, group 3 in green, and group 4 in pink.
